# Digital pills: a scoping review of the empirical literature and analysis of the ethical aspects

**DOI:** 10.1186/s12910-019-0443-1

**Published:** 2020-01-08

**Authors:** Andrea Martani, Lester Darryl Geneviève, Christopher Poppe, Carlo Casonato, Tenzin Wangmo

**Affiliations:** 1 Institute for Biomedical Ethics, University of Basel, Bernoullistrasse 28, Basel, Switzerland; 2 Faculty of Law, University of Trento, Trento, Italy

**Keywords:** Digital pills, Digital medicine, Mobile health, Monitoring devices, Scoping review, Ethics of technology

## Abstract

**Background:**

Digital Pills (DP) are an innovative drug-device technology that permits to combine traditional medications with a monitoring system that automatically records data about medication adherence as well as patients’ physiological data. Although DP are a promising innovation in the field of digital medicine, their use has also raised a number of ethical concerns. These ethical concerns, however, have been expressed principally from a theoretical perspective, whereas an ethical analysis with a more empirically oriented approach is lacking. There is also a lack of clarity about the empirical evidence available concerning the application of this innovative digital medicine.

**Methods:**

To map the studies where DP have been tested on patients and discuss the ethically relevant issues evident therein, we performed a scoping review of the empirical literature concerning DP.

**Results:**

Our search allowed us to identify 18 papers reporting on studies where DP were tested on patients. These included studies with different designs and involving patients with a variety of conditions. In the empirical literature, a number of issues with ethical relevance were evident. At the patient level, the ethical issues include users’ interaction with DP, personal sphere, health-related risks and patients’ benefits. At the provider level, ethically relevant issues touch upon the doctor-patient relationship and the question of data access. At the societal level, they concern the benefits to society, the quality of evidence and the dichotomy device-medicine.

**Conclusions:**

We conclude that evidence concerning DP is not robust and that more research should be performed and study results made available to evaluate this digital medicine. Moreover, our analysis of the ethically relevant aspects within empirical literature underscores that there are concrete and specific open questions that should be tackled in the ethical discussion about this new technological solution.

## Background

Healthcare is becoming a data intensive environment where a huge amount of data is both produced and consumed [1]. In this context, digital medicine is assuming an increasingly important role [2, 3]. Differently from *digital health*, a broad term that encompasses all those technical solutions related to health and medicine, such as telemedicine or electronic health records [4], the meaning of digital medicine is narrower. Specifically, digital medicine refers to “those products that are undergoing rigorous clinical validation and/or that ultimately will have a direct impact on diagnosing, preventing, monitoring or treating a disease, condition or syndrome” [5]. Digital medicine includes a wide range of devices, such as temperature-monitoring foot mats capable of automatically detecting diabetic foot ulcers or clinically validated smartphone apps for smoking cessation combined with video tutorials and nicotine replacement therapy [3]. These products share some features with traditional medications – such as the fact that they need approval from regulatory bodies before accessing the market – but they also differ from them. In fact, unlike standard medicines, the functioning of several digital medicine products relies primarily on technological elements, rather then – for instance – on new active principles, in an attempt to combine innovative technology with traditional therapy or medication [6] in what has been also defined as the emerging field of “digital therapeutics” [7].

One of the most recent and advanced technological medication developed in the field of digital medicine are digital pills (DP). DP are drug-device combinations that collect and transmit individual measurement data from patients both in the clinical and the research setting to monitor some health-related lifestyle habits and, in particular, medication-taking behaviour [8]. DP are comprised of three complementary elements: an ingestible sensor, a wearable patch and a mobile application connected to an external web server. The ingestible sensor is a small digital marker that – after being ingested by patients – is activated by the acid fluids in the stomach and releases a signal detectable by the wearable patch. The wearable patch is a plaster applied to the abdomen of the patient that records not only data about the occurred ingestion transmitted by the digital marker, but also other physiological data – such as heartbeat and daily steps. All information collected through the wearable patch is automatically transmitted to an application installed on the patients’ phone. The application then uploads the data on a web-based portal, which makes it potentially accessible to the patient herself, as well as her family, and her healthcare providers. DP have been designed to integrate traditional drugs, in that the ingestible sensor can be co-encapsulated with normal medicines to allow a reliable monitoring of medication-taking behaviour and the collection of data concerning other health-related lifestyle habits [8, 9].

DP have been recognised “as a qualified method for measuring adherence in clinical trials” by an opinion of the European Medicines Agency (EMA) in 2016 [10] and at the end of 2017 the first DP combined with a traditional drug was granted market approval as a medication by the Food and Drug Administration (FDA) in the US [11]. The first DP approved as a medication consists in a combination of this device with aripiprazole, a drug to treat mental illnesses such as schizophrenia or bipolar disorder. The review process by the FDA evaluated the evidence produced by the DP developers and decided for approval arguing that “if the [..] system fails, patients will not incur additional risk; they will continue to receive the exact treatment benefits of aripiprazole tablets without tracking. If the system works as intended and the patient chooses to share the data with the HCP [health care providers], the drug ingestion data could potentially help guide the prescribing physician on treatment interventions” [12].

After the first approval of a DP combined with a traditional drug, it is foreseeable that many other traditional medications will be *digitalised*. In particular, the developers of DP argue that digitalisation of traditional drugs would be particularly useful for the treatment of chronic illnesses, such as Type 2 diabetes, hypertension, Alzheimer’s disease and hepatitis C [13]. In fact, medication-taking behaviour is often suboptimal for patients with such chronic illnesses, and finding solutions to help tackle this problem would entail both better health outcomes and great savings in terms of healthcare resources [14]. In this respect, DP have been described as a landmark advancement, since they would “pioneer a path toward improving the quality and cost of care for the millions of people suffering from uncontrolled illness” [15]. Moreover, it has been observed that DP could help improve the communication and the counselling interventions of healthcare providers thanks to the possibility of transmitting real-time reliable data about patients and their health-related behaviour [16].

The idea that traditional drugs are integrated into DP in order to automatically collect and share patients’ data has also generated a great number of ethical concerns. It has been argued that collecting data through DP might affect individuals’ autonomy [17], represent an unpleasant form of surveillance [18], introduce elements of coercion in the treatment of patients [19], impact on the doctor-patient relationship [18, 20], compromise privacy [21] and over-enhance the idea of responsibility for health [22]. Some authors have even compared taking DP to “swallowing a spy”, which would collect and upload a huge amount of sensitive data without bringing any substantial therapeutic benefit to the patients [23]. Others consider DP as a potential first step towards a biomedical “big brother” [24].

Although the ethical issues that the use of DP might generate are extensively discussed, the literature providing ethical analysis of DP is predominantly theoretical in its nature, whereas an ethical reflection based directly on the data emerging from studies where DP have been tested is lacking. With the objective to complement the existing theoretical literature, the purpose of this scoping review is twofold. Firstly, it maps published empirical studies where DP have been tested with patients, in order to provide an overview of the available empirical evidence on this digital medicine. Secondly, it provides - in the context of those studies - a discussion on the ethics of this digital medicine based on the data from the studies were DP were tested.

## Methods

To conduct this scoping review, we followed the methodological framework elaborated by Arksey and O’Malley [25] and updated by Levac et al. [26]. We also followed the recently published PRISMA-ScR checklist for reporting of scoping reviews [27].

Within this framework, our questions were:

*“What empirical research has been done where DP have been tested to collect patients’ data?*

*What, if any, ethically relevant aspects concerning the use of this digital medicine are evident in that empirical research?”*

As recommended by Levac et al. [26], these questions are both broad and focused. They are broad in that they address the growing and diverse body of literature concerning DP (i.e. we decided not to limit our quest to the in-patient or out-patient setting or to the use of DP in combination with one specific traditional drug). They are also focused, in that they are limited to empirical research and considered only studies involving patients (i.e. no healthy volunteers). We were aware that much literature of a theoretical nature had been published concerning ethical issues related to DP [28]. However, we decided to focus our research question only on empirical literature since our objective was to ground an ethical analysis on the published empirical studies where DP were tested.

### Search strategy

**Fig. 1:**
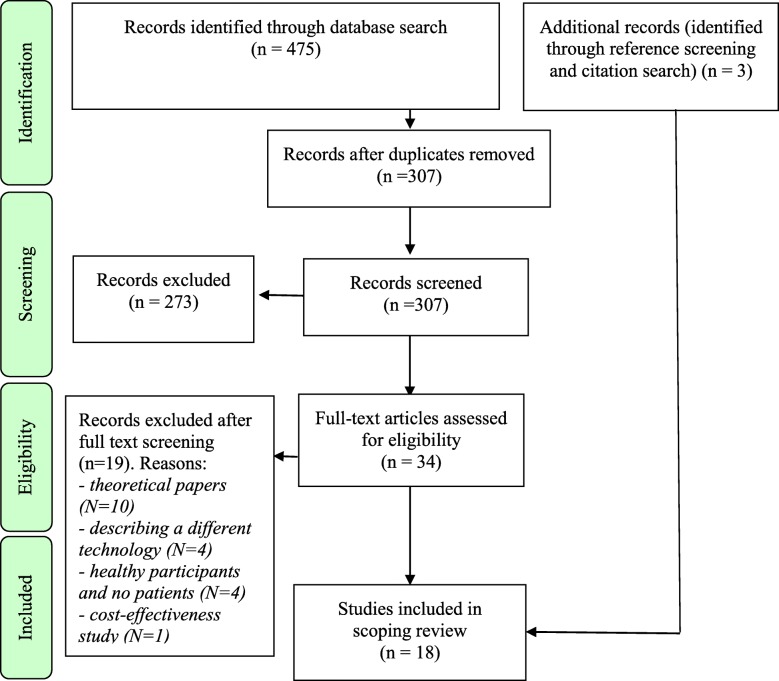
PRISMA flowchart of study selection

In order to retrieve the relevant studies where DP were used in the patient setting, we performed a literature search through four search engines, namely PubMed, CINAHL, Scopus (MEDLINE) and Embase.^1^ We built our search strategy as broad as possible to find all the studies that combined the two main subject fields of our quest, namely the DP technology and the context of data collection. We limited our literature search to publications from 2010 onwards, since – through a preliminary search of the literature – we observed that the first DP prototype received official certification of safety and quality only then [29]. Our literature search was conducted on 05/09/2018 and produced 475 results (see Fig. 1).

### Study selection

Study selection was divided into three steps, as recommended by Peters et al. [30]. Firstly, duplicates were eliminated, thus reducing the number of records from 475 to 307. Secondly, a preliminary screening based on title and abstract was performed independently by two authors. After the independent abstract screening stage, there was an initial discordance with respect to which records to exclude on 33 papers (10.75%), which was solved through debate until consensus was reached. It was finally agreed to exclude 273 records, either because they were conference abstracts or they did not concern DP. The 34 records remaining after abstract screening underwent full-text assessment for eligibility, which was performed independently by AM, CP and LDG. In this case, the concordance rate between different assessors was 100%. Consequently, additional records (*n* = 19) were excluded, either because they were theoretical papers not reporting on empirical studies with patients (*n* = 10), or because they were describing a different digital medicine technology (*n* = 4), or because they involved only healthy participants (*n* = 4), or because they were cost-effectiveness studies based on hypothetical data (*n* = 1). The final number of included records resulting from our literature search (*n* = 15) was then complemented by additional papers retrieved via reference screening (*n* = 2) and citation search (*n* = 1). In the end, the definitive number of records wherefrom data were extracted consisted of 18 papers.

### Data charting

For data charting, we decided to be comprehensive and extract both general data about the characteristics of the included studies (e.g. type of intervention, study population) and then information concerning which ethically relevant aspects were evident to the assessors. Every included record was analysed independently by two authors to enhance the accuracy and completeness of data extraction. Data concerning the general characteristics of the studies were recorded according to a data extraction form based on the PICO model, adapted to the specific features of the present review, which included studies with quite different designs. In order to chart data concerning ethically relevant aspects from the studies, we started from the framework developed by Klugman et al. [6]. In their theoretical study concerning digital medicine, Klugman et al. [6] hypothesized that ethical aspects related to DP and similar technologies can be of three natures, namely patient-related, provider-related, and society-related. Although within this framework they also provided a list of ethically relevant issues, we decided not to be bound by their framework in extracting data, since Klugman et al. [6] admit their list is only tentative. We adopted a bottom-up approach and searched for all those aspects in the included records that had an ethical dimension of a patient-related, provider-related or society-related nature. To ensure comprehensiveness and reliability of data extraction with this bottom-up approach, we met and preliminary discussed what could constitute an ethically relevant aspect. After two authors examined each paper independently, they coded all the information that they individually considered of ethical relevance. The authors then met another time to crosscheck the data they coded, they organised them according to themes and justified why they considered the different themes of ethical relevance based on a connection with one of the principle of biomedical ethics [31]. When disagreement emerged, this was solved through debate until consensus was reached. A summary of the reasoning behind the choice of themes and the justification why they were considered of ethical relevance is provided in the Additional file 2. The authors then refined the specific themes and sub-themes to organise, collate and then report the ethically relevant aspects retrieved from the analysed records. These were then ordered in the categories defined by Klugman et al. [6].

## Results

### General features of the included studied

This scoping review resulted in the analysis of 18 papers reporting on studies where DP were used to collect individual data from patients. Table 1 illustrates and summarises the general features of the included records.

**Table 1:** Overview of the features of the studies

Paper ID^a^	Country	Study design	Study population	Aim(s) of the study	REC/IRB^b^ Approval
Au-yeung 2011 [32]	USA	3 prospective observational studies	30 patients with Tuberculosis, 8 with Heart Failure, 43 with Hypertension^c^	Evaluate the system and characterize technical performance.	Yes
Belknap 2013 [33]	USA	Feasibility study: prospective and observational.	30 patients with active tuberculosis (TB)	Evaluate accuracy, safety and acceptability of the system.	Yes
Browne 2015 [34]	USA	Prospective observational cohort-study	5 patients with type II diabetes	Characterize the at-home adherence patterns of patients through modern methods of visual analytics.	Yes
Browne 2018 [35]	USA	Randomized cross-over study	12 patients with active tuberculosis	Address Good Manufacturing Practice methods to combine the ingestion sensor with oral medications.	Yes
Chai 2017a [36]^d^	USA	Prospective descriptive study	16 patient with acute fractures^e^	Report data on opioid ingestion patterns detected by DP	Yes
Chai 2017b [37]^d^	USA	Pilot study: Prospective, non-randomized and observational.	10 patients with acute fractures	Determine the feasibility the digital pill system to measure opioid ingestion patterns	Yes
Dicarlo 2016 [38]	USA	Feasibility study: prospective, non-randomized, observational.	37 patients with hypertension	Record patterns of medication-taking, step count, daily blood pressure and weight. Study safety and acceptability of digital pills	Yes
Eisenberg 2013 [39]	Switzerland	Exploratory study: open-label, non-randomised and prospective.	20 patients after kidney transplant under Enteric-coated mycophenolate sodium (ECMPS)	Evaluate the detection accuracy, usability, and safety of DP combined with ECMPS in kidney transplants.	Yes
Frias 2017 [40]	USA	Pilot study: prospective, open-label, cluster-randomized (three arms).	109 adults with uncontrolled Hypertension and type II diabetes	Study the effect of digital pills on blood pressure, glycemic and lipid control, engagement, and provider decision making.	Yes
Kane 2013 [41]	USA	Pilot study: observational, non randomised.	28 subjects with schizophrenia (16) or bipolar disorder (12)	Compare the detection accuracy to that of a directly observed method. Characterise safety and user satisfaction.	Yes
Kopelowicz 2017 [42]	USA	Pilot study: observational, open-label and non-randomised.	49 subjects with bipolar disorder, major depressive disorder, or schizophrenia	Evaluate the functionality of an integrated call center in optimizing the use of the digital pills and assess its use.	Yes
Moorhead 2017 [43]	USA	Post hoc studies based on a study following a cluster randomised design.	113 patients with uncontrolled hypertension.	Study the incremental impact of seeing versus not seeing DP medication dose reminders on medication-taking and assess the safety of the digital pills with respect to possible risk of overdosing.	Yes
Naik 2017 [44]	UK	Prospective registry-based observational study.	151 patients with uncontrolled hypertension^f^	Characterize patterns of medication use. Assess usability and acceptability of digital pills.	Yes
Noble 2016 [45]	UK	Prospective observational study.	39 patients with uncontrolled hypertension	Report and summarise the first use of digital pills by pharmacists to establish blood pressure management recommendations.	Not required
Peters-strickland 2016 [46]	USA	Phase II open-label observational study.	67 patients with schizophrenia	Assess the usability of the system, satisfaction, safety and tolerability.	Yes
Peters-strickland 2018 [47]	USA	Six formative human factors studies	129 patients with confirmed diagnosis of schizophrenia, bipolar I disorder, or major depressive disorder (MDD)	Assess the safe and effective use of a system. Assess whether the three intended groups of users (patients, healthcare providers, and caregivers) can appropriately use the technology.	Yes
Rohatagi 2016 [48]	USA	Phase 4 exploratory observational study: open-label and single-arm.	58 stable patients with a diagnosis of bipolar I disorder (*n* = 35) or MDD (*n* = 23)	Obtain descriptive feedback from patients, assess safety and summarize patient adherence	Yes
Thompson 2017 [49]	UK	Prospective observational study	21 patients with either established cardiovascular disease or high multifactorial risk	Test the system in a group of patients at elevated cardiovascular risk attending a cardiac prevention and rehabilitation program	N/A

^a^ First author and publication year

^b^ Research Ethics Committee (REC) or Institutional Review Board (IRB)

^c^Only 40 completed the study

^d^ this study used a version of the ingestible sensor and wearable patch produced by a different company

^e^ Only 15 patients completed the study

^f^ 167 patients were enrolled in the registry, but 16 were excluded from the study

Apart from three studies conducted in the UK and one in Switzerland, the great majority of the studies (*n* = 14) took place in the United States.

In terms of study design, one group (*n* = 14) of the studies were prospective and observational. Within this group, six studies were further described as “pilot” (*n* = 4) or “feasibility” (*n* = 2) studies, two terms that normally refer to trials which are conceived as preparatory to larger confirmatory studies [50]. Two more of this group were additionally described as exploratory, which also refers to studies with a strong tentative component. The remaining studies outside the prospective/observational group (n = 4) had slightly different designs. One was a prospective and descriptive study not offering any specific analysis of the data it produced. The others were a randomised cross-over study, a post-hoc study and a human factors study.

The included studies tested DP with patients having a wide range of conditions or illnesses. In total, the 18 studies included 896 participants ranging from 5 [34] to 151 [44]. Six studies included patients with uncontrolled hypertension, where DP were co-encapsulated with different types of traditional medications belonging, for example, to the category of beta-blockers or angiotensin-converting-enzyme inhibitors. In five studies, the patient population was comprised of patients with psychiatric disorders, such as schizophrenia, bipolar disorder or depression. In these cases, DP were used in combination with antipsychotic medication, principally aripiprazole. Three studies included patients with tuberculosis (TB) and in these cases DP were combined with TB medications, such as isoniazid and rifampin. In two studies, DP were tested with patients suffering from acute fractures and they were used together with opioid medications. Two studies included patients suffering from type II diabetes, and DP were combined with metformin or sulfonylurea. Two studies also addressed patients with cardiovascular problems and DP were used in combination with furosemide or other cardiovascular medications. In the only study where the patient population consisted of patients having received kidney transplant, DP were combined with Enteric-coated mycophenolate sodium.^2^

In terms of objectives, the studies had different specific purposes, but they were normally aimed at exploring different features of the DP system, its acceptability and its accuracy. Only one compared traditional therapy with DP therapy [40], since participants were cluster randomised in three groups, one with traditional care and two with DP medications. Only one study [49] compared explicitly the accuracy of the DP in monitoring medication-taking behaviour in comparison with self-reporting by the patient.

Almost every study (*n* = 16) was approved either by a REC or an IRB. One study [45] reported that in its case ethics approval was not needed. One study [49] did not report any information concerning ethical review or approval. In many studies (*n* = 14), at least one author was an employee or had a conflict of interest to declare concerning his relationship with the producers or developers of DP.

### Ethically relevant aspects

**Fig. 2:**
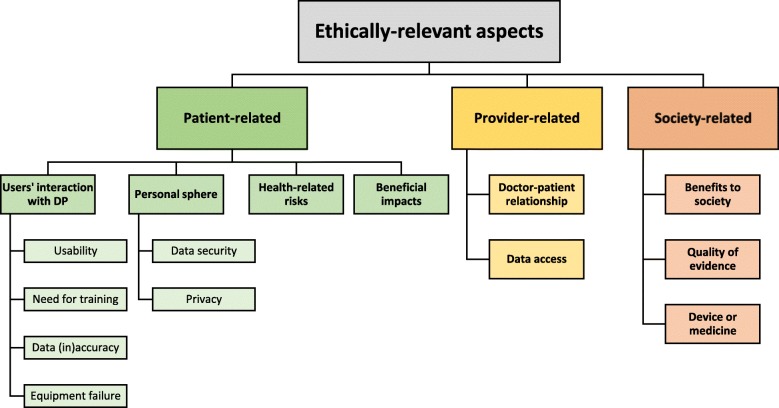
Ethically relevant aspects

Within the included records (*n* = 18), a wide range of ethically relevant aspects were extracted and presented in Fig. 2. As recommended by the methodological framework for conducting scoping reviews [25] and also often made in scoping reviews with an ethical scope [51], results are reported in a narrative fashion.^3^

### Patient-related

Patient-related aspects are widely mentioned in the analysed records and they include considerations about the interaction between DP and patients, issues concerning patients’ personal sphere, health-related risks and benefits of DP at the individual level.

Considerations about the interaction between DP and patients touch on different topics. Many papers (*n* = 10) reflect on the usability of DP and generally underscore that patients give positive feedback concerning the functional aspects of this technology. For instance, one study [44] claims that “ninety-two percent (92%) of patients reported that they did not mind wearing the wearable sensor. More than 87% of patients reported having a good experience from using the ingestible [*sic*] and thought that it was easy to understand and convenient to use”. Ten papers explicitly report that using DP requires some form of training for patients, who, on top of indications concerning the medicine they are taking, also need to learn how to operate the wearable patch and the mobile application. For example, one study [46] reports that “the patients received structured training at baseline (BL) and additional direct weekly support/remedial training” and that “optimal performance [of DP] depends on continuous use of the wearable sensor, which requires a patient’s ability to regularly replace the sensor and pair it each time with a smartphone application”. Another important issue concerning the interaction between users and this digital medicine is whether DP data accurately mirror patients’ behaviour. Part of the records (*n* = 7) tackle the issue of the accuracy of the data recorded through DP. Generally, it is underscored that the system is accurate, either by reporting how precise DP are in registering the occurred ingestion or by underscoring that false positives (i.e. DP recording ingestions although these have not occurred) were rare. Lastly, some other papers (*n* = 7) tackle another relevant factor to consider for patients in their interaction with DP, namely the possibility to experience equipment failure. One paper, for example, stresses that data can be successfully transmitted only if the user’s phone has signal [37]. Or else, in another paper [42] it is underlined that data transmission can occur successfully only if patients regularly keep their phone near them. Another study [49] also underlines the necessity for patients of contacting technical support to troubleshoot connectivity issues.

Reflections about patients’ personal sphere in the studies touched upon the themes of data security and privacy. Almost all the records (*n* = 14) explain that data collection and data transmission are safe and secure, mentioning, for example, that personal information is encrypted [38] or that a secure server is used [39]. Some papers (*n* = 9) also refer to privacy considerations, by underlining the vast amount of personal data that are collected [34], by reporting that these data are collected in a private way as data transmission is confined to the body of patients [32], who are anyway fully aware of being monitored [44]. In one study testing DP with opioids medication given to patients suffering from acute fractures [37], it is argued that “formative interviews of study participants demonstrate their perception that the digital pill maintains patient privacy. [ …] This suggests that individuals with other stigmatized conditions [ …] may also similarly accept the data security offered through the digital pill”. One study [41] underlines that, despite the close monitoring of patients’ activities, “no subjects developed new onset of paranoid ideation”.

The last types of ethically relevant aspects at the individual level evident in the studies are health-related risks and the benefits of DP to the health of patients. Almost all records (*n* = 14) address issues related to potential health-risks. The majority of them simply report the adverse effects of DP, which are mostly related to local skin irritation produced by the wearable patch. For example, one study [39] underscores that “2 [patients discontinued treatment prematurely] due to skin intolerance to the APM [adhesive personal monitor, i.e. the wearable patch]” and another study [46] that “there were five TEAEs [treatment-emergent adverse events] (rash, papular rash, rash pruritic, pruritus, skin discoloration) that led to study discontinuation of four patients.” Only one study [34] reports that there were no adverse events related to DP usage. One study [47] hints at the possibility that “use errors [ …] might lead to a patient taking a one-pill extra dose”, but then claims that these cases “were rare in the validation study”. In terms of the beneficial impacts of DP, only a minority of the studies (*n* = 8) report them and considerations are quite disparate and cautious. For example, one study [43] says that its “findings demonstrated improved medication taking for patients”, but only when these “are near their mobile device during medication dose times”. Another study [40] underlines that “participants [who used DP] had significantly greater reductions in SBP [systolic blood pressure] within 4 weeks than the usual care group” and thus DP “can help patients improve their level of BP and diabetes control”.

### Provider-related

Two typologies of provider-related ethically relevant issues are addressed in the studies, namely the impact of DP on the doctor-patient relationship and the question of data access (i.e. who – and on what conditions – can monitor patients’ data collected through DP).

Ethically relevant issues concerning the relation between healthcare professionals and patients are addressed in several studies (*n* = 14). Some studies highlight that DP could foster patient self-care and self-management. For example, one study [43] says how DP “continuously engages with patients about the pharmacologic and non-pharmacologic therapies of their treatment plan”; another study [38] underlines that thanks to DP “digital health data [ …] are passively acquired to be provided automatically to patients as part of ongoing feedback on their health behaviors”. Other studies, however, claim that DP require more interventions and more interaction with medical professionals since “participants preferred real-time transfer of ingestion data to their physician, especially if their physician could use their ingestion data to intervene at potential times of escalating use [of the medicine]” [37]. Other studies claim that DP are adaptable in this respect, since “assistance from a caregiver was allowed; however, patients were encouraged to use the DMS [acronym for DP] independently” [42]. Eight studies also insist on the idea that DP allow healthcare providers to receive data concerning medication adherence that is “actual” or “objective”, in contrast to other methods relying on patients’ information (e.g. self-reporting). For example, one study [42], although not providing any direct evidence as to the accuracy of data collected through DP, claims that DP “addresses daily adherence by detecting and registering the ingestion of *actual* doses taken by a patient, it provides an *alternative* and *objective* means of closely managing medication therapy to ensure adherence and optimal outcomes” (emphasis added). Another study [36] stresses that “digital pills [ …] can provide *direct* and *definitive* evidence of medication ingestion” (emphasis added).

Part of the papers (*n* = 8) also deal with the issue of data access and discuss who can monitor patients’ data and at which conditions. In all these cases, studies underline that the patient is in control of data collected through DP and that she is the only one who can determine who else (e.g. family members or healthcare providers) may access it. For example one study [32] states that “fundamentally, the information gathered by the networked system belongs to the patient user; he or she has the right to determine whether or not and with whom to share this information.”

### Society-related

As far as the societal level is concerned, three types of ethically relevant issues are present. Firstly, almost the entirety of studies (*n* = 14) mention that DP might bring about considerable societal benefits. Some (*n* = 11) mention how DP could improve prescribing practices and medication-taking. For example, one study [45] underscores that “the information that is collected [ …] can be used to determine the root cause for uncontrolled hypertension during existing antihypertensive treatment, thereby providing an evidence base for appropriate prescribing recommendations, and a means for avoiding the medicine wastage”. Some others (*n* = 3) highlight that DP can help rationalise the use of healthcare resources. For example, it is reported that “the future application of the system could allow more efficient use of resources, particularly personnel” [33]. Lastly, it is hypothesised that DP might contribute to the implementation of individualised medicine (*n* = 7). For instance, one study [43] highlights that with DP “health care providers can view patient data with the use of a patient portal, facilitating more targeted treatment and lifestyle recommendations”.

Secondly, a considerable number of studies (*n* = 11) reflect on the limited quality of the evidence they produce concerning DP, due to small sample size and lack of generalizability. For example one study [46] describes that “most of the enrolled patients were male and black and were rated as mildly ill [ …] and all were capable of using the smartphone; therefore, the current results may not be generalizable to a more typical population of patients with schizophrenia”. Or else, one other study [33] reckons that “the sample size for this feasibility study was small and larger studies are needed to further document the sensitivity, specificity, usability, acceptability, and cost-effectiveness of the system”.

Some records (*n* = 8) mention a third important issue at the societal level, i.e. they refer to the fact that the device-components of DP had already received official approval as medical devices, although DP not having yet been approved as medicines, i.e. when used in combination with a traditional drug for curative purposes. In fact, the first versions of the components of DP (i.e. the ingestible marker and the wearable patch) received market approval as class IIa medical devices in 2010 in the EU and they received FDA clearance in 2012 in the US [38]. On the contrary, the first DP used in combination with a traditional drug only received approval in 2017 for the US market.

## Discussion

This scoping review offers an overview of published literature where DP have been tested with patients and presents systematically the ethically relevant aspects. The first important finding is that published studies are quite diverse in their design, but are predominantly explorative, non-randomised and with small numbers of participants. This suggests that the evidence publicly available concerning DP is not robust. Indeed, the lack of rigorous control and double-blind methodology in studies testing digital medicine have been described as problematic, since it exposes the validity of research results to both the placebo effect (i.e. the psychological impact of knowing to be taking the medication) and the Hawthorne effect (i.e. the impact of the observer/researcher on the behaviour of participants) [52]. Moreover, small sample sizes and flexibility in study designs have been described as two important factors that affect substantially the validity of results [53]. It is also surprising that, although some studies explicitly test the accuracy of DP (i.e. whether the device correctly records data about medication-taking), only one of them [49] somehow compares the accuracy of DP with that of another method for assessing medication-taking, namely self-reporting. More evidence in this respect would be particularly important since, even if DP are admittedly not aimed at guaranteeing better medication adherence [54], they nevertheless constitute a system that claims to objectively monitor medication-taking behaviour. From an ethical standpoint, comparing the accuracy of DP in monitoring medication-taking behaviour with that of other traditional methods (e.g. pill counts, self-reporting) would be very important, since it could be a convincing reason to justify the closer digital surveillance and the higher privacy risks that DP entail. Another important finding is the absence of studies on specific age groups, which might have their particular features and present different sets of challenges. For example, focusing on young adults and adolescents might reveal that this age group has aesthetical concerns related to DP, something which has been observed with respect to other body devices for diabetes monitoring and treatment [55].

One further relevant finding that emerges from our results is that DP have been tested in combination with patients suffering from different illnesses. Such variety certainly demonstrates that DP may be applied in different contexts, but it also reveals that little research has been published with respect to the use of DP with every single illness. Although the core elements of this digital medicine remain the same (i.e. they monitor medication-taking behaviour), it cannot be presumed that findings concerning the use of DP with one specific illness could be equally valid for other types of diseases. Every illness presents patients and doctors with different challenges and it is should be studied more in details how DP affect those diverse situations. Moreover, we found only one study [40] that compares DP therapy including a *digitalised* traditional medication with the *non digitalised* therapy. Accurately testing whether the *digitalised* version of a medicine has better outcomes in terms of medication adherence than its *non digitalised* variant would be quite important from an ethical point of view, since it could help decide if there is a substantial interest, for example in terms of beneficence, for society to shift from traditional to *digitalised* medications.

With respect to the ethically-relevant aspects at the patient level, our results indicate that many issues related to the personal sphere of patients, especially with respect to data security, are evident in the empirical literature concerning DP. In this respect, there are two important findings to point out. First, our results show that DP are designed in a way to ensure data security and to protect privacy. However, results also suggest that the protection of the personal sphere is reduced to something that concerns mainly technical aspects (e.g. encryption, security of servers). On the contrary, the moral dimension of privacy, which is particularly relevant in the field of health data management [56], remains underappreciated. DP allow to monitor delicate aspects of people’s lives – such as the fact that they are taking medications for mental disorders – thus requiring that also aspects of privacy other than data-security are considered – such as potential loss of control over the intimate sphere and disempowerment. This is particularly important to decide on the possible future use of DP in the regular clinical context. In the latter case, protecting privacy might not be achieved simply by encrypting the data, but it would require also making sure that patients do not feel indirectly coerced to opt for DP – for example if health insurances expected the use of digitalised medications as a condition to cover treatment costs or employers as a guarantee that workers are preserving their health [57]. Second, the emphasis on privacy being protected at the technical level through encryption or the use of secure servers cannot overshadow the fact that personal aspects of private life are nevertheless monitored and thus potentially accessible to other parties. In this respect, it must be underlined that DP also collects lifestyle data, thus potentially exposing other personal behaviour. Only one study [37] reports that its participants did not express concerns about privacy in a less technically oriented meaning, but the study in question did not involve directly the use of DP for the treatment of chronic or stigmatising conditions. Moreover, another study [41] marginally mentions that the close monitoring of patients seems not to be linked with the development of paranoid feelings, but it does not explore in details the potential links between surveillance through DP and paranoia. Investigating this aspect would be particularly important, since – as outlined above – the clinical use of DP involves also the treatment of psychiatric disorders such as schizophrenia.

Our results further reveal that studies acknowledge how DP create a few complications for their users. The system is not always accurate because it depends on some external factors (e.g. that the battery of the wearable patch is charged) and it requires training. Considering these elements is relevant from an ethical perspective, since their impact – for example in terms of quality of life – needs to be evaluated to determine whether DP are truly beneficial for patients. If the promise of DP is to be “smart” [58], then more evidence is needed to verify whether the complications that are related to their technological components do not end up constituting a burden for patients.

Our results at the provider level reveal that two inconsistencies related to the use of DP emerge from the literature. The first concerns the doctor-patient relationship. On the one hand, studies discuss that the objective of DP is to foster self-care and reduce dependency on (and contacts with) healthcare professionals. On the other hand, it is also claimed that DP would require more interventions by health professionals, whose role seems to be idealised – as if they were practically capable of constantly monitoring patients’ medication-taking data, to then promptly intervene whenever needed [18]. The presence of this dichotomy suggests that it is not clear whether DP entail an increase or a reduction of workload for doctors in the everyday provision of care. In either case, it is important to ensure that monitoring devices like DP do not compromise the communication between patients and medical professionals, and the support the latter can offer in the implementation of any treatment plans. The second inconsistency concerns data access. By looking at the ethically relevant aspects at the provider level it emerges that many studies regard as an essential element that control over data access is retained by patients, so that they can decide freely whether data can be shared with other parties (e.g. family members or healthcare providers). At the societal level, however, studies argue that the advancements in terms of societal beneficence – such as providing individualised care or improving medication-taking behaviour – are essentially dependent on the sharing of data between patients and other subjects. None of the records discusses how the apparent contradiction between these two claims – that patients have free choice whether they want to share their data and that societal benefits require sharing of data – can be resolved.

With respect to the societal level, the most relevant results concern, again, the quality of the empirical evidence available of DP studies. As noted by Vayena and Ienca [59], an essential element for the ethical assessment of digital medicines is the presence of significant empirical evidence concerning their functioning, their benefits and their risks. In particular, it is important that preliminary studies of a device have representative samples whose results are generalizable and have external validity, so that they can be assumed to apply to the wider public that will make use of the technology after its approval [60]. Our results show that the great majority of published DP studies acknowledge significant limitations related to small sample sizes and thus to the generalizability of results. This indicates that comprehensive evidence to thoroughly assess, for example, if the extensive use of DP can improve the cost-effectiveness of certain treatments in a given healthcare context, is lacking.

Moreover, results concerning the societal level raise two further important issues. Firstly, the question emerges whether the use of DP is beneficial from the collective perspective. In the included studies, there is emphasis on the societal benefits achievable through the digitalisation of traditional medicines. However, no concrete evidence in this sense is produced directly by the studies themselves. Societal benefits are simply mentioned as hypothetical and future outcomes of the extensive use of DP. To truly prove the cost-effectiveness of DP, a comparison would have to be made between the traditional version of a medication and its corresponded digitalised form, both in terms of outcomes and of costs [61]. During our literature search, we had retrieved one cost-comparison study concerning DP [62], but we excluded it since its findings were based on calculations using hypothetical data. The second issue concerns the repetitive mentioning of the approval of the technological components of DP as medical devices. This underlines that a societal reflection is needed to decide whether to keep this existing dichotomy, where a much different path of approval exists for medical devices in comparison to drugs. The first one is much less restrictive and it is comparable to the type of approval of washing machines, lawn mowers or videogames consoles [63]. Moreover, possibilities exist in some states to further reduce governmental control concerning approval for market access if medical devices are deemed to be substantially equivalent to previously cleared devices – such as the often criticised 510 (k) process in the US [64]. Given the profound impact on many aspects of treatment that digital devices like the ones included in DP can have, this reinforces the existing claim that the approval process of this kind of devices should be modernised [65].

### Limitations

Limitations of this review include the fact that the search strategy was limited to some databases and that the relatively newness of this digital medicine entails a terminological unevenness amongst publications which makes it difficult to capture all the published work. Even DP – the name chosen for this manuscript – is not uniformly used throughout the literature. However, the fact that we complemented our initial search not only by screening the references, but also by citation search gives us confidence about some level of completeness of our review among published data. We nevertheless acknowledge that – due to the nature of the approval process for digital medicines – there could be other DP studies that are not published and hence unavailable to the team. Another limitation is the way we gathered ethically relevant issues from the included papers, which could be subjective. To increase transparency of our decision making process, we have explained our rationale in the Additional file 2. Lastly, being our focus on published work of an empirical nature, we have not included all the literature of a theoretical nature, where many ethical issues concerning DP have been thoroughly discussed, and unpublished work. Companies developing DP might not publish some of the studies they have conducted in academic journals and the authors have no resources to contact companies and get such information. Yet, the purposes of this review were indeed to explore empirical literature concerning DP and ground an ethical analysis in the elements evident directly therein, in an attempt to bridge the gap between literature reporting on studies where DP were actually tested and the theoretical literature on this digital medicine.

## Conclusions

DP represent an example how digital medicine - and indeed more in general the application of technology to the field of healthcare – is a complex and divisive field of enquiry, where enthusiasm for innovation and diffidence from the ethical perspective clash. To help overcome this deadlock with respect to DP, this review has provided more clarity about the content of empirical research currently available. It has presented an overview of the empirical literature on DP and has mapped the ethically relevant issues mentioned therein, in order to discuss some aspects of new technology with a less theoretical approach. This sets the basis for future research and discussions concerning both the potential and the concerns related to a wider use of DP and the digitalisation of traditional drugs.

## Supplementary information

**Additional File 1.** Search strategy implemented in a search string for PubMed.

**Additional File 2.** List of Themes and justification of their ethical relevance.

**Additional File 3.** List of Coded Sources and Themes.

## Data Availability

All the data were retrieved from articles from publicly available databases and it is presented in the tables and the additional files of this manuscript. The review protocol and the data extraction form are available from the corresponding author on reasonable request.

## Abbreviations

DP: Digital Pills
ECMPS: Enteric-coated mycophenolate sodium
EMA: European Medicines Agency
FDA: Food and Drug Administration
IRB: Institutional Review Board
MDD: Major depressive disorder
REC: Research Ethics Committee
TB: Tuberculosis

## Footnotes

1 An example of how the search strategy was implemented in a source string to be used in the databases can be found in the supplementary material [Additional file 1].

2 Some records contained details about more than one study and some considered more than one illness.

3 A detailed depiction of the distribution of ethically relevant issues – clearly organised article per article – is available in the Additional file 3.
