# Supplementary material for: Digital pills: a scoping review of the empirical literature and analysis of the ethical aspects
Source: BMC Med Ethics. 2020 Jan 8;21:3. doi: 10.1186/s12910-019-0443-1 (PMC6950823; doi:10.1186/s12910-019-0443-1)
Supplement: Supplementary file 1 — Additional File 1. Search strategy implemented in a search string for PubMed. [file 12910_2019_443_MOESM1_ESM.docx]

**Additional file 1.**

**Search strategy implemented in a search string for PubMed**

(("digital" AND "medicine offering") OR ("smart pill") OR (digital health feedback system[tw]) OR ("digital" AND "adherence-assessment" AND "device") OR ("ingestible" AND "sensor") OR ("Ingestion event" AND "monitoring") OR ("sensor enabled" AND "medicine*") OR ("Abilify" AND "MyCite") OR ("proteus" AND "digital health") OR ("drug-device" AND "combination") OR (“digital” AND “pill”)

AND

("data" OR "information" OR "measur*" OR "collect*" OR "record*" OR "monitor*" OR "detect" OR "register*")
