# Supplementary material for: Digital pills: a scoping review of the empirical literature and analysis of the ethical aspects
Source: BMC Med Ethics. 2020 Jan 8;21:3. doi: 10.1186/s12910-019-0443-1 (PMC6950823; doi:10.1186/s12910-019-0443-1)
Supplement: Supplementary file 2 — Additional File 2. List of Themes and justification of their ethical relevance. [file 12910_2019_443_MOESM2_ESM.docx]

# **Additional File 2. List of Themes and justification of their ethical relevance.**

| **Ethically relevant issue^a^** | **Domain to which it is pertinent** | **Bioethical principles to which it is connected** | **Reason for considering this an “ethically relevant issue”** |
| --- | --- | --- | --- |
| Beneficial impacts | Patient-related | Beneficence | The “beneficial impacts” - that are listed or mentioned in the studies - help define what the advantages for patients who use DP are. Put it differently, highlighting the beneficial impacts contributes to answer the question: “What is the benefit for patients if they use DP?” |
| Benefits to society | Society-related | Justice | “Benefits to society” are linked to justice since the adoption of a new technology in the field of medicine is also addressed to solve problems at the collective level, especially concerning better use of resources. For example, in the case of DP, one of the reasons why they were developed is to tackle poor medication-adherence, which is causing severe problems at a societal level, especially due to waste of resources. With this issue, we wanted to look for insights to the question: “What does society stands to benefit from the introduction of DP?” |
| Data access | Provider-related | Autonomy | We concluded that considerations about the access to the data that patients produce by ingesting DP affect patients’ autonomy, in that they reveal information about their behaviour to the healthcare provider. In other words, issues concerning data access are relevant to autonomy since they determine to what extent patient’s health related behaviour is monitored and can also be steered. |
| Data (in)accuracy | Patient-related | Beneficence | The level of accuracy of one of the main functions of DP (namely monitoring medicine taking behaviour) is quite an important issue concerning the beneficence for the patient. In fact, “how accurate DP are” is a factor that helps to determine the overall benefit that patients might derive from using them. |
| Data security | Patient-related | Autonomy | “Data security” is closely linked to autonomy. The data DP collect (mediation taking behaviour and other lifestyle data such as heartbeat and steps made) are sensitive health-related data. The level of security and the measures taken to achieve it, are thus two important elements to determine how these data about the intimate sphere of the patient are safeguarded from potential intrusion of third non-authorised parties. |
| Device or medicine | Society-related | Justice | Issues concerning the approval of DP by a public authority (e.g. FDA) are important to reflect on societal questions concerning appropriateness of the procedures used to approve these product. This is particularly interesting for a technology such as DP, which are a medical device to be integrated with a drug (thus – to some extent – blurring the boundaries between these two categories). Moreover, the process of approval of a medical device or a drug – as a matter of fact – determine the level of safety and effectiveness that society deems acceptable to allow a product on the market, which might vary with time and with technological advancement. |
| Doctor-patient relationship | Provider-related | Beneficence | At the same time the “doctor patient relationship” also concerns the principle of beneficence. For example, if DP reduce the number of visits to GPs, this might have a negative impact on the welfare of the patient. At the same time, reducing visits to GPs might also have positive impacts if – for example – this reduction requires less travelling to the healthcare facility and the quality (and length) of the fewer visits is improved. |
| Equipment failure | Patient-related | Non-maleficence | “Equipment failure” is an issue that plays an important role with respect to the principle of non-maleficence. In fact, if a device is *per se* accurate and effective, but at the same time subjects to malfunctions, this might result damaging to the well-being of the patient. |
| Health-related risks | Patient-related | Non-maleficence | “Health related risks”, especially in terms of potential adverse reactions, are relevant to consider for the principle of non-maleficence to avoid negative health outcomes for patients. |
| Need for training | Patient-related | Autonomy | The presence or absence of a “need for training” might have an impact on autonomy at least in two ways. On the one hand, more training needed means that patients require more support in operating the device and then exercise their autonomy with respect to the treatment. On the other day, the presence of training might also help patients expand their autonomy, in that after the training they might become less dependent on, for example, their doctor. |
| Privacy | Patient-related | Autonomy | “Privacy” is strictly related to autonomy, since the intrusion in the personal and intimate sphere of a patients impacts on his freedom to act freely – especially with respect to the healthcare provider. |
| Quality of evidence | Society-related | Justice | The “quality of the evidence” concerning a the performance of a health technology is a key feature to reflect, for example, whether that technology should be financed in the publicly-funded health system, or if can be used by health insurances to adjust coverage. |
| Usability | Patient-related | Beneficence | “Usability” is closely linked to beneficence, since a device that is non-user-friendly can compromise its usefulness for the patients and impact on patients’ welfare. |

^a^ alphabetical order
