# Supplementary material for: Digital pills: a scoping review of the empirical literature and analysis of the ethical aspects
Source: BMC Med Ethics. 2020 Jan 8;21:3. doi: 10.1186/s12910-019-0443-1 (PMC6950823; doi:10.1186/s12910-019-0443-1)
Supplement: Supplementary file 3 — Additional File 3. List of Coded Sources and Themes. [file 12910_2019_443_MOESM3_ESM.docx]

# **Additional File 3. List of Coded Sources and Themes, By Source**

# **Patient-related ethically relevant issues**

| **Included records^a^** | **Usability** | **NEED FOR TRAINING** | **DATA INACCURACY** | **eQUIPMENT FAILURE** | **DATA SECURITY** | **privacy** | **Health related risks** | **beneficial impacts** |
| --- | --- | --- | --- | --- | --- | --- | --- | --- |
| Au-yeung2011 [30] |  |  | X |  | X | X | X | X |
| Belknap2013 [31] | X |  | X |  | X | X | X |  |
| Browne2015 [32] |  |  |  | X | X | X |  |  |
| Browne2018 [33] |  |  | X |  | X |  | X | X |
| Chai2017a [34] | X | X | X | X |  |  |  |  |
| Chai2017b [35] | X | X | X | X | X | X | X |  |
| Dicarlo2016 [36] |  | X |  |  | X |  | X | X |
| Eisenberg2013 [37] |  | X | X | X | X | X | X |  |
| Frias2017 [38] | X | X |  |  |  | X | X | X |
| Kane2013 [39] | X | X | X |  | X | X | X |  |
| Kopelowicz2017 [40] |  |  |  | X | X | X | X | X |
| Moorhead2017 [41] |  | X |  |  | X |  |  | X |
| Naik2017 [42] | X |  |  |  | X | X | X | X |
| Noble2016 [43] |  |  |  |  | X |  |  | X |
| Peters-strickland2016 [44] | X | X |  |  | X |  | X |  |
| Peters-strickland2018 [45] | X | X |  |  | X |  | X |  |
| Rohatagi2016 [46] | X |  |  | X |  |  | X |  |
| Thompson2017 [47] | X | X |  | X |  |  | X |  |
| **TOTAL** | **10** | **10** | **7** | **7** | **14** | **9** | **14** | **8** |

^a^ first author and year of publication

# **Provider-related ethically relevant issues**

| **Included records^a^** | **Doctor-patient relatonship** | **Access to data** |
| --- | --- | --- |
| Au-yeung2011 [30] | X | X |
| Belknap2013 [31] |  |  |
| Browne2015 [32] | X |  |
| Browne2018 [33] |  | X |
| Chai2017a [34] | X |  |
| Chai2017b [35] | X |  |
| Dicarlo2016 [36] | X |  |
| Eisenberg2013 [37] | X |  |
| Frias2017 [38] | X |  |
| Kane2013 [39] |  |  |
| Kopelowicz2017 [40] | X | X |
| Moorhead2017 [41] | X | X |
| Naik2017 [42] |  | X |
| Noble2016 [43] | X | X |
| Peters-strickland2016 [44] | X |  |
| Peters-strickland2018 [45] | X |  |
| Rohatagi2016 [46] | X | X |
| Thompson2017 [47] | X | X |
| **TOTAL** | **14** | **8** |

^a^ first author and year of publication

# **Society-related ethically relevant issues**

| **Included records^a^** | **benefits to society** | **quality of evidence** | **device or medicine** |
| --- | --- | --- | --- |
| Au-yeung2011 [30] | X | X |  |
| Belknap2013 [31] | X | X |  |
| Browne2015 [32] | X | X | X |
| Browne2018 [33] | X |  | X |
| Chai2017a [34] | X | X | X |
| Chai2017b [35] | X | X |  |
| carlo2016 [36] | X |  | X |
| Eisenberg2013 [37] | X |  | X |
| Frias2017 [38] | X | X |  |
| Kane2013 [39] | X | X | X |
| Kopelowicz2017 [40] |  |  |  |
| Moorhead2017 [41] | X | X |  |
| Naik2017 [42] | X |  | X |
| Noble2016 [43] | X |  | X |
| Peters-strickland2016 [44] |  | X |  |
| Peters-strickland2018 [45] |  | X |  |
| Rohatagi2016 [46] | X |  |  |
| Thompson2017 [47] |  | X |  |
| **TOTAL** | **14** | **11** | **8** |

^a^ first author and year of publication
